# Short video addiction scale for middle school students: development and initial validation

**DOI:** 10.1038/s41598-025-92138-x

**Published:** 2025-03-22

**Authors:** Jianmei Ye, Weijun Wang, Dawei Huang, Shihao Ma, Shuna Chen, Wanghao Dong, Xin Zhao

**Affiliations:** 1 Key Laboratory of Adolescent Cyberpsychology and Behavior (CCNU), Ministry of Education, Wuhan, 430079 China; 2 School of Psychology, Central China Normal University, Wuhan, 430079 China; 3 Key Laboratory of Human Development and Mental Health of Hubei Province, Wuhan, 430079 China; 4 School of Humanities and Management, Guizhou University of Traditional Chinese Medicine, Guiyang, 550025 China; 5 Manchester Institute of Education, The University of Manchester, Manchester, UK; 6 Institute of Digital Commerce, Wuhan Technology and Business University, Wuhan, 430065 China

**Keywords:** Short video addiction, Adolescents, Middle school students, Scale development, Psychometric validation, Psychology, Human behaviour

## Abstract

The rise of short video platforms has increased concerns about addiction, especially among adolescents. This study aimed to develop a Short Video Addiction Scale for middle school students. A sample of 1492 middle school students participated in the study. Initial items were derived from qualitative interviews and refined through psychometric analyses, including Exploratory and Confirmatory Factor Analyses. The final scale, consisting of 15 items across five factors (Academic Procrastination, Interpersonal Strain, Social Communication Difficulties, Attention Concentration Difficulties, and Impaired Control over Short Video Use), demonstrated high internal consistency (Cronbach’s α = 0.900), test-retest reliability, and strong validity. The Short Video Addiction Scale, a 15-item scale, demonstrates robust psychometric properties. It is reliable and valid for assessing short video addiction among middle school students and will be a valuable tool for identifying and addressing the growing concerns of short video addiction in adolescents.

**Supplementary Information:**

The online version contains supplementary material available at 10.1038/s41598-025-92138-x.

## Introduction

In the current digital era, with the rapid rise of short video platforms and the widespread adoption of intelligent internet devices, the number of short video users in China has reached 1.053 billion, accounting for 96.4% of the overall internet user base. Among them, the number of adolescent internet users is nearly 200 million^1^. Short video platforms have become the most commonly used apps among minors, with a usage rate as high as 65.3%. Notably, middle school students exhibit the highest usage rate of these apps, at 67.9%^2,3^. This indicates that middle school students have become the primary users of short video apps within the adolescent demographic. Short videos, defined as clips under 5 min in length, are characterized by their lightweight nature, everyday content, interactivity, immersive experience, and ease of dissemination^4^. The hedonistic characteristics of short videos attract excessive use among users^5,6^. Consequently, short video addiction has emerged as a new public health issue^7^.

Short video addiction refers to problematic behavior where individuals engage in compulsive or excessive viewing of short videos, leading to significant behavioral dysregulation or attention deficits, and subsequently causing difficulties in interpersonal relationships, academic performance, and/or work adaptation^8^. The negative impact is particularly pronounced among middle school students. This stage represents a critical period in adolescence, marking the transition from childhood to adulthood. During this time, individuals undergo rapid physical maturation, cognitive development, self-identity exploration, changing social needs, academic challenges, and the formation of morals and values, all while facing a heightened risk of mental health issues^9^. During this stage, middle school students’ self-control abilities are still immature, making them highly susceptible to problems related to excessive use of short videos, which can lead to consequences such as increased depression, feelings of loneliness, attention deficits, poor sleep quality, and social isolation^10^. In addition, addiction to short videos can adversely affect middle school students’ motivation and learning abilities, leading to diminished academic aspirations^11^ and avoidance behaviors in studying^12^. Hence, conducting research on short video addiction among middle school students is crucial for timely detection and intervention of problematic behaviors, thereby promoting healthy development among adolescents.

However, despite the rapid rise and development of short video platforms, academia has yet to establish universally recognized standards for measuring short video addiction. Previous studies have measured short video addiction using scales directly adapted from internet addiction^13,14^, mobile phone addiction^5^, social media addiction^10,15^, Facebook addiction^16,17^, social network service addiction^6,18^, and gaming addiction^19,20^. For instance, Aykut Günlü and colleagues^21^ adapted the Instagram Addiction Scale to develop the Scale for Measuring Problematic TikTok. This scale assesses the level of TikTok addiction among Turkish adults, encompassing three factors: the dimensions of obsession, escapism, and lack of control. Qin^22^ referenced Young’s theory of internet addiction^23^ and adapted a mobile phone addiction scale to develop a short video addiction scale for college students. Studies by Bai^24^ and Luo^25^ have also developed Short Video Addiction Scale for College students through qualitative research methods. However, Bai and Luo’s study only assessed internal consistency reliability, lacking test-retest reliability and construct validity. Furthermore, their research targeted college students, which limits its applicability for measuring short video addiction among middle school students. Further examination is needed to establish the scale’s reliability and validity.

In summary, while existing short video addiction scales have been adapted from other addiction behavior scales, they exhibit notable limitations in reliability and validity, particularly when applied to the unique context of short video addiction. These scales have been found to be inadequate for capturing the distinct characteristics of short video addiction, which are shaped by the rapid pace, visual appeal, and interactive nature of this medium. Moreover, a critical oversight in existing scales is their failure to account for the psychological developmental characteristics specific to middle school students. The identified flaws in existing scales include a lack of age-appropriate content, insufficient consideration of the social and cultural influences that are prevalent among adolescents, and an absence of items that reflect the specific cognitive and emotional processes of middle school students. These limitations are not merely a matter of insufficient data on the validity of existing scales; rather, they represent fundamental gaps that prevent these scales from accurately measuring short video addiction in our target demographic.

To address these serious flaws, this study is designed to develop a novel scale through qualitative research that deeply explores the psychological traits and behavioral patterns of middle school students. Our aim is to create a measurement tool—Short Video Addiction Scale for Middle School Students (SVAS-MSS) that is not only more applicable and targeted but also scientifically robust in assessing the phenomenon of short video addiction among this demographic. This tailored scale will enable us to promptly identify potential issues, support relevant prevention and intervention efforts, and ultimately promote the healthy development of middle school students by providing a more nuanced understanding of short video addiction.

### STUDY 1: Qualitative research on short video addiction among middle school students

Study 1 seeks to comprehensively explore the psychological and behavioral manifestations of short video addiction among middle school students, along with its effects on their academic performance and daily functioning. This investigation serves as the foundation for developing an initial item pool for a scale specifically designed to measure short video addiction in this population.

### Interview design

This study adopts a semi-structured interview approach to examine the psychological and behavioral responses of middle school students following their exposure to short videos. Based on Dong et al.’s (2023) definition, short video addiction is characterized by compulsive viewing that leads to uncontrolled behaviors or attention deficits, which, in turn, disrupt interpersonal relationships, academic performance, or work adaptation. Given that middle school students’ lives predominantly revolve around school, the interview topics are largely school-centered.

In line with this definition, the core themes of the interview are categorized into five key areas:

1. Psychological and behavioral manifestations of compulsive short video viewing;
2. Uncontrolled behaviors induced by short video usage;
3. Attention disorders caused by short video usage;
4. Interpersonal relationship difficulties triggered by short video usage;
5. Learning adaptation challenges resulting from short video usage.

The interview outline was structured around these themes (see Supplementary Material 1). During interviews, the lead investigator may expand on relevant questions depending on the participant’s responses and the flow of the interview to gather more in-depth insights.

### Participants and procedure

*Sample 1: Qualitative Analysis.* This study utilized a snowball sampling method for interviews, with data collection concluding upon reaching saturation. To account for potential regional differences, the sample included 16 junior high school students from eastern, central, and western China (9 females, 7 males; 4 in Grade 7, 3 in Grade 8, and 9 in Grade 9; age range 12–15 years, *M* = 13.30, *SD* = 0.978). Additionally, two Grade 8 teachers, including both homeroom and subject teachers, were interviewed.

Informed consent was obtained from both participants and their parents, with signed consent forms. A trained psychology graduate student conducted the interviews, clarifying that the data would be used solely for research purposes and that all personal information would remain confidential. Participants were compensated 15 RMB upon completion of the interviews.

### Collection and analysis of interview data

#### Data collection and organization

Given the geographic diversity of participants from eastern, central, and western China, all interviews were conducted via telephone. Pre-interviews were first conducted with five junior high school students to refine the final interview outline. Thereafter, interviews with both students and secondary school teachers continued until data saturation was reached. Each interview was transcribed into Chinese within 24 h, with both the original recordings and preliminary transcripts preserved. To ensure accuracy, researchers repeatedly listened to the recordings during transcription, eliminating irrelevant or incoherent content. For privacy, the data of the 16 students and 2 teachers were anonymized and labeled as M1 through M18.

### Data encoding

This study employed Nvivo12 to analyze the interview transcripts from Sample 1 (totaling 65,519 words), focusing on the five core themes outlined in the interview guide for coding and node statistics. Three graduate students in psychology conducted the coding process, identifying 156 nodes related to short video addiction symptoms. The analysis involved three levels of coding: 155 primary codes, 14 secondary codes, and 5 tertiary codes, categorized into the following dimensions: interpersonal issues (24 nodes), academic adaptation difficulties (43 nodes), attention deficits (27 nodes), behavioral control problems (48 nodes), and compulsive viewing (13 nodes). Detailed examples of the coding process are provided in Supplementary Material 2.

### Data analysis results

Interview findings demonstrate that short video addiction among middle school students is characterized by five prominent dimensions: interpersonal relationship difficulties, challenges in academic adaptation, attention concentration impairments, Impaired Control over Short Video Use, and compulsive video consumption. Specifically:

*Firstly, Interpersonal Relationship Impairments*: Addiction to short-form video content leads to a reduction in communication between junior high students and their parents, subsequently triggering frequent disputes and strained familial relationships. Interview findings indicate that excessive consumption of short videos diminishes the time allocated for parent-child interactions and exacerbates family conflicts. Simultaneously, although the sharing of short videos can facilitate the development of friendships among peers, the inappropriate or excessive use of internet memes (hot memes) may undermine relationships with classmates. This dual impact not only induces tension within the family unit but also fosters misunderstandings and conflicts within peer relationships, thereby comprehensively deteriorating the quality of students’ interpersonal interactions.

*Secondly, Challenges in Academic Adaptation*: The engagement with short-form video content undermines students’ capacity to complete assignments in a timely manner, thereby precipitating challenges in academic adaptation. While some students utilize short videos as a mechanism for stress alleviation, this practice consumes excessive time, resulting in insufficient study periods and exacerbated academic pressure, thereby fostering a deleterious feedback loop. Viewing short videos during nocturnal hours impairs attentiveness in subsequent classroom sessions, attenuating the efficacy of instructional delivery. Interview findings reveal that post-viewing consumption of short videos leads to a decline in cognitive functions and memory retention among students, adversely impacting academic performance. Furthermore, procrastination behaviors exacerbate these challenges, as students exhibit difficulty in regulating their viewing habits, resulting in the postponement or extension of homework completion times. This compromises their time management capabilities, heightens psychological strain, and ultimately culminates in a decline in academic achievement.

*Thirdly, Attention Concentration Difficulties*: Prolonged engagement with short-form video content results in narrowed attention spans among students, hindering their ability to concentrate on external stimuli. After viewing short videos, students frequently revisit the content during classes or while completing assignments, leading to dispersed attention. The inherent characteristics of short videos—namely their brevity, rapid pacing, and substantial information density—diminish students’ patience for extended tasks such as studying and homework, thereby weakening their sustained attention. Moreover, addiction to short videos among junior high students not only reduces their focus on real-world interpersonal interactions but also impairs their capacity to maintain concentration due to the recurrent recollection of video content. These factors collectively undermine academic performance and efficiency.

*Fourth, Behavioral Loss of Control*: Short video platforms employ algorithms that incessantly curate content aligned with students’ interests, thereby impeding their ability to regulate viewing behaviors and relinquish their mobile devices. In real-life contexts, students frequently emulate actions observed in videos or incorporate internet memes into their interactions, manifesting signs of impaired behavioral control. This phenomenon not only disrupts daily routines but also precipitates behavioral deviations. Furthermore, attempts by students to curtail or cease short video consumption often prove unsuccessful, rendering them unable to interrupt viewing sessions and subsequently hindering their academic and personal schedules. Consequently, addiction to short videos markedly diminishes self-regulatory capacities, adversely affecting both daily functioning and academic performance.

*Fifth, Compulsive Viewing*: A subset of students report an inability to discontinue short video consumption despite a lack of desire or awareness of its detrimental effects, indicative of persistent compulsive behavior. This relentless engagement imposes substantial mental stress and psychological burdens. Additionally, attempts to reduce or discontinue viewing frequently result in failure, leading to increased psychological strain and emotional fatigue. The continuous nature of compulsive viewing not only undermines academic performance and interpersonal relationships but also exerts profound negative impacts on mental health, thereby impeding students’ ability to overcome short video addiction.

In summary, the interview findings validate the definition of short video addiction and demonstrate its multifaceted impacts across five distinct domains. Furthermore, they reveal the negative effects on students’ family and peer relationships, academic management, attention concentration, behavioral regulation, and mental health. These results not only elucidate the profound influence of short video addiction on the daily lives and academic performance of junior high students but also provide a critical foundation for the development of a short video addiction scale tailored to this population.

### STUDY 2: Development and psychometric evaluation of a SVAD-MSS

Study 2 builds on the findings from Study 1, aiming to develop a psychometrically sound scale for assessing short video addiction among middle school students.

### Participants and procedure

Before administering the questionnaire, informed consent was obtained from participating schools, parents, and students. Both parents and students who agreed to participate provided written consent. Mental health education teachers conducted the testing sessions, emphasizing that the data would be used exclusively for research purposes and that personal information would remain confidential. Participants were informed of their right to withdraw at any time without consequences. Upon survey completion, participants received a gift worth 2 RMB and corresponding practice credits.

*Sample 2: Exploratory Factor Analysis.* A convenience sampling method was used to select a school in central China. Six classes were randomly selected, totaling 343 participants. After excluding 21 invalid responses, 322 valid questionnaires were retained, yielding a 93.8% effective rate. The sample included 120 first-year, 109 s-year, and 93 third-year students, with 177 males and 145 females, ages ranging from 12 to 16 years (*M* = 13.37, *SD* = 1.056).

*Sample 3: Test-Retest Reliability.* Two weeks later, a subset of 157 participants from Sample 2 was selected. After excluding 14 invalid responses, 143 valid questionnaires remained, with a 91.1% response rate. The retest response rate of 91.1% is slightly lower than the initial testing (sample 2) response rate of 93.8%. This minor decrease in response rate may indicate a small degree of participant drop-off between the two testing phases; however, both rates are high, suggesting a strong level of engagement from the participants across both stages of the study. It is also important to note that the ratio of participants from the second (retest) testing to participants from the first testing is 157/343, which is approximately 45.8% of the original sample. This indicates that a considerable proportion of the original participants were involved in the retest, further supporting the reliability of the findings.

The sample 3 included 91 first-year and 52 s-year students, with 78 males and 65 females, ages ranging from 12 to 14 years (*M* = 12.85, *SD* = 0.768).

*Sample 4: Confirmatory Factor Analysis, Reliability, Validity Testing, and Measurement Invariance.* To ensure sample diversity, schools from eastern, central, and western China were selected. It is important to note that there was no overlap between this sample and samples 2/3, ensuring distinct participant pools for each phase of the study. The breakdown of valid responses was as follows: Eastern Region: 279 (effective rate: 89.7%), Central Region: 236 (effective rate: 93.3%), and Western Region: 514 (effective rate: 96.62%). Overall, 1009 valid questionnaires were collected, consisting of 486 first-year and 523 s-year students, with 554 males and 455 females, ages ranging from 11 to 15 years (*M* = 12.93, *SD* = 0.645).

### Items development

Based on qualitative analysis and coding results, the preliminary **SVAD-MSS** was developed, encompassing the following dimensions and items. Interpersonal Relationship Issues: This dimension includes difficulties with friends and parental relationships (13 items). Academic Adaptation Issues: Addressing impacts on class engagement, academic performance, learning skills, and procrastination (17 items). Attention Deficits: Covering reduced focus, difficulty shifting attention, and narrowed attention (11 items). Behavioral and Emotional Dysregulation: Including loss of control over usage time, imitating behaviors, and emotional instability (10 items). Compulsive Viewing: Encompassing compulsive behavior and thinking (4 items).

The preliminary scale initially contained 54 items. An expert panel, comprising one psychology professor, six psychology doctoral students, and four psychology master’s students, reviewed the items. Based on their feedback, 15 items were removed due to redundancy and ambiguity, and others were revised for clarity, resulting in a final version of 39 items (9 for interpersonal relationship issues, 12 for academic adaptation issues, 6 for attention deficits, 8 for Impaired Control over Short Video Use, and 4 for compulsive viewing).

To ensure clarity and reduce potential misunderstandings, three middle school students reviewed the items. Feedback led to revisions where necessary, resulting in the finalized 39-item scale. The items are detailed in Supplementary Material 3.

### Measures

*Preliminary SVAD-MSS.* The preliminary **SVAD-MSS** developed in this study consists of 39 items across five dimensions: interpersonal issues, academic adjustment problems, attention difficulties, Impaired Control over Short Video Use, and compulsive viewing, all related to short video use. The items are framed as first-person statements, such as “In conversations with family/friends/classmates, I have ignored what they said because I was engrossed in watching short videos.” Responses are measured on a 5-point Likert scale, with options ranging from “Never” (1) to “Always” (5). Higher scores reflect a greater level of short video addiction.

*Smartphone Addiction Scale-Short Version*,*** SAS-SV.*** The scale referenced is the SAS-SV developed by Kwon et al.^26^. It consists of 10 items measuring a single dimension of smartphone addiction, using a 6-point Likert scale. Higher scores indicate a greater degree of smartphone addiction. Kwon et al. established the cutoff scores for smartphone addiction as higher than 33 for females and 31 for males. In this study, the Cronbach’s α coefficient for the SAS-SV was 0.905, indicating high reliability.

*Social Networking Site Addiction (SNS Addiction) Scale.* This scale is the Facebook Intrusion Questionnaire (FIQ) developed by Elphinston et al.^27^. It includes 8 items and uses a 7-point Likert scale, with higher scores indicating a greater risk of social network addiction. The Cronbach’s α coefficient for the FIQ in this study was 0.830, demonstrating good reliability.

*The Academic Stress Questionnaire for Middle School Students.* This scale was used, which was developed by Xu et al.^28^. This questionnaire includes four dimensions: parental pressure, self-pressure, teacher pressure, and social pressure. This scale consists of 21 items, using a Likert scale of 5 points, with higher total scores indicating a higher level of academic stress for the individual. The Cronbach’s α coefficient for this questionnaire in this study is 0.877, indicating a high level of reliability.

*The Academic Burnout Questionnaire.* This scale was adopted from the version revised and translated by Luo et al.^29^. The revised Chinese version consists of 15 items, covering three dimensions: emotional exhaustion, depersonalization, and a sense of low achievement. This scale comprises 15 items, using a 4-point Likert scale, with higher scores indicating a greater degree of academic burnout. The Cronbach’s α coefficient for this questionnaire in this study is 0.795, indicating a high level of reliability.

### Statistical analyses

The data were processed using SPSS 27 and Mplus 8.0. First, the interview content from Sample 1 was analyzed to develop the preliminary version of the scale. For Sample 2, item analysis and exploratory factor analysis were conducted. Items with low total correlations, low factor loadings, or cross-loadings were removed, resulting in the finalized scale.

Subsequently, Mplus 8.0 was used to perform confirmatory factor analysis on the data from Sample 4. Goodness-of-fit indices were used to evaluate the models^30^: Comparative Fit Index (CFI; ≥ 0.90), Tucker-Lewis Index (TLI; ≥ 0.90), and Root-Mean-Square Error of Approximation (RMSEA; ≤ 0.08) with a 90% confidence interval. Reliability and validity were assessed using SPSS 27, while Mplus 8.0 was used to test the scale’s measurement invariance. Test-retest reliability was evaluated using data from Sample 3.

### Ethics

All procedures in this study adhered to the Declaration of Helsinki and were approved by the Ethics Committee of Central China Normal University. Participants were fully informed about the study’s scope and provided written informed consent.

## Results

### Item analysis

Combined with the conceptual definition of short video addiction and the results of qualitative analysis, the factor structure of the SVAD-MSS is latent multidimensional constructs. To assess the quality of the items in the preliminary SVAD-MSS, item-total correlation analysis was conducted for Sample 2 (*n* = 322) to evaluate item discrimination. Items with correlation coefficients below 0.4 were excluded to ensure high homogeneity across the scale. The results of the item-total correlation analysis are presented in Table 1. Based on the data, the following low-homogeneity items were removed: 1, 6, 7, 11, 14, 24, 27, 28, 34, and 35, resulting in the exclusion of 10 items.

**Table 1:** Item-total correlation coefficients for the preliminary short video addiction scale (*n* = 322).

Item	Correlation coefficient	Item	Correlation coefficient	Item	Correlation coefficient	Item	Correlation coefficient
**1**	0.275***	**11**	0.375***	21	0.574***	31	0.560***
2	0.535***	12	0.500***	22	0.618***	32	0.446***
3	0.530***	13	0.460***	23	0.589***	33	0.527***
4	0.572***	**14**	0.340***	**24**	0.167***	**34**	0.382***
5	0.571***	15	0.583***	25	0.481***	**35**	0.374***
**6**	0.355***	16	0.603***	26	0.600***	36	0.480***
**7**	0.369***	17	0.543***	**27**	0.388***	37	0.590***
8	0.453***	18	0.579***	**28**	0.256***	38	0.625***
9	0.491***	19	0.523***	29	0.420***	39	0.564***
10	0.594***	20	0.512***	30	0.505***		

*p < 0.05, **p < 0.01, ***p < 0.001. Items in bold are those marked for deletion.

### Exploratory factor analysis

To explore the factor structure of the initial SVAD-MSS, exploratory factor analysis (EFA) was conducted on Sample 2.

First, based on the item analysis, the Kaiser-Meyer-Olkin (KMO) measure of sampling adequacy and Bartlett’s test of sphericity were performed on the remaining 29 items. The KMO value was 0.912, and Bartlett’s test yielded a chi-square value of 3974.744 (*df* = 378, *p* < 0.001), indicating that the data were suitable for EFA.

**Fig. 1:**
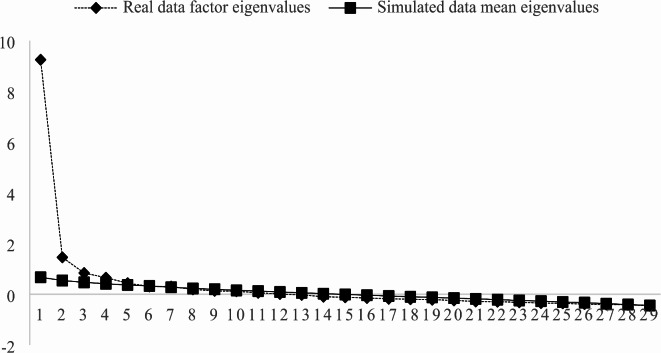
Scree plot of parallel analysis.

Next, parallel analysis with principal axis factoring and oblique rotation was used to extract factor loadings, the result retained five effective factors (see Fig. 1). The initial exploratory factor analysis results are presented in Table 2.

**Table 2:** Initial results of exploratory factor analysis (*n* = 322).

Item	Factor1	Factor2	Factor3	Factor4	Factor5	Factor6	Communality
3	0.970						0.633
33	0.702						0.455
4	0.660						0.53
**5**	0.407				0.346		0.485
**2**	0.362		0.315				0.407
**9**	0.323						0.319
**30**							0.332
36		0.716					0.500
31		0.704					0.542
32		0.662					0.449
29		0.591					0.358
**25**		0.531					0.433
**21**		0.443		0.339			0.556
20			0.788				0.531
12			0.768				0.505
**17**			0.508	0.347			0.476
**23**			0.48		0.309		0.512
16			0.445				0.492
18				0.694			0.626
22				0.622			0.623
26				0.515			0.558
39				0.353	0.655		0.500
38					0.598		0.554
**15**				0.379	0.439		0.557
**19**			0.329		0.398		0.377
**37**							0.402
8						0.837	0.572
**13**			0.308			0.372	0.385
**10**						0.348	0.439

Coefficients less than 0.3 are not shown. Items marked in bold are to be deleted.

After the initial analysis, items were removed sequentially based on the following criteria in the specified order:

- *Communalities < 0.3*: No items were excluded, as all items met or exceeded this threshold.
- *Factor loadings < 0.4*: Seven items (2, 9, 10, 13, 19, 30, 37) were eliminated.
- *Cross-loadings (loading > 0.4 on multiple factors with < 0.3 difference between loadings)*: Five items (5, 15, 17, 21, 23) were removed.
- *Factors with fewer than 3 items*: After applying criteria 1–3, Factor 5 initially fell below the 3-item threshold. However, items 38 and 39 were retained due to their high representativeness, resulting in no further exclusions.
- *Poor interpretability*: One item (25) was removed for thematic inconsistency with Factor 2.

The sequential exclusion process ensured that earlier criteria (e.g., factor loadings, cross-loadings) influenced subsequent decisions (e.g., retaining Factor 5 despite its reduced item count). Ultimately, five factors were retained, explaining 68.964% of the total variance. Post-rotation factor loadings, communalities, eigenvalues, and contribution rates are detailed in Table 3.

**Table 3:** Final results of exploratory factor analysis (*n* = 322).

Item	Factor1	Factor2	Factor3	Factor4	Factor5	Communality
31. Watching short videos has made me increasingly impatient with other activities (such as conversations, reading, etc.).	0.779					0.622
32. While talking with family/friends/classmates, I ignore what they are saying because I am busy watching short videos.	0.773					0.664
36. I have reduced communication with my family/friends/classmates because of watching short videos.	0.763					0.616
29. I feel that my language expression ability has declined due to watching short videos.	0.692					0.554
3. I delay starting my homework because of watching short videos.		0.917				0.764
33. I watch short videos while doing homework, which prolongs the time needed to complete it.		0.806				0.664
4. I spend more time watching short videos than I initially planned.		0.691				0.601
18. After watching short videos, I find it hard to focus on other tasks.			0.908			0.804
22. After watching short videos, I find it difficult to refocus on studying.			0.884			0.725
26. Watching short videos makes it hard for me to concentrate on tasks that require prolonged thinking or studying.			0.478			0.692
12. My parents/friends/classmates think that my academic performance has declined because of watching short videos.				0.908		0.769
20. My parents/friends have expressed dissatisfaction or complaints because I watch short videos.				0.884		0.765
16. I have argued with my parents/friends because of watching short videos.				0.478		0.582
39. I have tried to control/stop my short video watching habits, but I have failed.					0.785	0.764
38. I cannot immediately stop watching short videos to do other things.					0.739	0.752
Eigenvalues after Rotation	5.658	1.484	1.245	1.126	0.832	
Contribution Rate	37.72%	9.89%	8.30%	7.51%	5.55%	
Cumulative Contribution Rate	37.72%	47.61%	55.91%	63.42%	68.96%	

Based on the analysis results, the final scale comprises 15 items divided into five factors (The specific items are detailed in Supplementary Material 4):

1. *Social Communication Difficulties*: It Comprises 4 Items, this factor assesses the negative impact of short video usage on students’ communication abilities with family members, friends, or classmates.
2. *Academic Procrastination*: It Comprises 3 items, this factor evaluates the academic procrastination behaviors resulting from short video usage.
3. *Attention Concentration Difficulties*: It Comprises 3 items, this factor reflects the difficulties students face in concentrating on other tasks or studies after watching short videos.
4. *Interpersonal Strain*: It Comprises 3 items, this factor measures the relational tension and conflicts with family and peers resulting from excessive short video usage.
5. *Impaired Control over Short Video Use*: It Comprises 2 items, this factor measures behaviors such as control failure and difficulties in immediate cessation during short video usage among students.

### Confirmatory factor analysis

To validate the structural validity of the SVAD-MSS, a Confirmatory Factor Analysis (CFA) was conducted on Sample 4. This study developed both a five-factor model and a higher-order factor model. The findings indicated that both models exhibited satisfactory fit indices (see Table 4). These results suggest that the overall score of the SVAS-MSS aligns with theoretical expectations and possesses significant practical relevance. Additionally, all standardized loadings ranged from moderate to high (loadings between 0.563 and 0.795), as detailed in Table 5. These indicators show that the five-factor model fits well, demonstrating that the scale has a relatively strong structural validity.

**Table 4:** Comparison of Five-Factor and Single-Factor models (*n* = 1009).

Model	*χ* ^2^	*df*	*χ*^2^/*df*	RMSEA	CFI	TLI	SRMR
Five-factor models	262	80	3.275	0.047	0.970	0.960	0.031
Higher-order factor models	297	85	3.494	0.050	0.965	0.956	0.033

**Table 5:** The standardized loadings of confirmatory factor analysis (*n* = 1009).

Factor	Item	The standardized loadings	Factor	Item	The standardized loadings
AP	3	0.782	IS	12	0.631
	4	0.777		16	0.623
	33	0.696		20	0.595
SCD	29	0.563	ACD	18	0.726
	31	0.724		22	0.761
	32	0.676		26	0.795
	36	0.665	ICSVD	38	0.779
				39	0.742

The abbreviations represent the following meanings: AP refers to Academic Procrastination; IS refers to Interpersonal Strain; SCD refers to Social Communication Difficulties; ACD refers to Attention Concentration Difficulties; and ICSVD refers to Impaired Control Over Short Video Use.

### Reliability analysis

To assess the reliability of the finalized the SVAS-MSS, this study analyzed both the Cronbach’s α coefficients and test-retest reliability.

#### Cronbach’s α coefficients

Using Sample 4, the Cronbach’s α coefficients for the scale were as follows: Total Scale = 0.900, Academic Procrastination = 0.791, Interpersonal Strain = 0.625, Social Communication Difficulties = 0.751, Attention Concentration Difficulties = 0.807, and Impaired Control over Short Video Use = 0.732. These results suggest that the scale and its subdimensions exhibit high internal consistency.

#### Test-retest reliability

Analysis was conducted using data from Sample 3. The test-retest reliabilities were as follows: Total Scale: 0.788; Academic Procrastination: 0.643; Interpersonal Strain: 0.602; Social Communication Difficulties: 0.614; Attention Concentration Difficulties: 0.622; Impaired Control over Short Video Use: 0.654. These findings indicate that the scale has acceptable test-retest reliability.

### Validity analyses

To assess the validity of the finalized the SVAS-MSS, this study examined its convergent validity, discriminant validity, and criterion validity.

#### Convergent and discriminant validity

Convergent validity was assessed using the Average Variance Extracted (AVE) and the Composite Reliability (CR) for each dimension (see Table 6). According to established standards, the AVE should exceed 0.4 and CR should exceed 0.6^31^. The results indicate that, with the exception of the AVE value for the “Interpersonal Strain” dimension, which is marginally below the threshold, all other dimensions meet the established criteria. Moreover, the CR values for all dimensions exceed 0.6. These findings collectively suggest that the scale exhibits acceptable convergent validity.

**Table 6:** Analysis results of convergent and discriminant validity (*n* = 1009).

	AP	IS	SCD	ACD	ICSVD
AP	–				
IS	0.486**	–			
SCD	0.525**	0.542**	–		
ACD	0.556**	0.526**	0.674**	–	
ICSVD	0.575**	0.452**	0.588**	0.624**	–
**AVE**	0.567	0.380	0.435	0.579	0.579
**√AVE**	0.753	0.616	0.660	0.761	0.761
**CR**	0.796**	0.647**	0.753**	0.805**	0.733**
**The correlation with the SVAS-MSS**	0.808**	0.738**	0.799**	0.834**	0.825**
**The highest correlation with other factors**	0.575**	0.542**	0.674**	0.674**	0.624**

*p<0.05, **p<0.01, ***p<0.001. The abbreviations represent the following meanings: AP refers to Academic Procrastination; IS refers to Interpersonal Strain; SCD refers to Social Communication Difficulties; ACD refers to Attention Concentration Difficulties; and ICSVD refers to Impaired Control Over Short Video Use.

To further assess the discriminant validity of the SVAS-MSS, the Fornell-Larcker criterion was employed^32^. According to this criterion, the square root of AVE (√AVE) for each factor should exceed the correlation coefficients between that factor and other factors (see Table 6). The results revealed that the correlations between all dimensions were statistically significant (*p* < 0.001). Notably, with the exception of the “Social Communication Difficulties” dimension, the square root values of the √AVE values for the remaining dimensions exceeded their respective correlation coefficients with other factors. This indicates that the SVAS-MSS exhibits acceptable discriminative validity.

This study examined the AVE and the √AVE between the SVAS-MSS and the criterion scales. The results, as presented in Table 7, indicate that the √AVE for the SVAS-MSS is 0.746, which exceeds its correlation coefficients with the Social Networking Site Addiction Scale (0.532), the Smartphone Addiction Scale (0.690), the Academic Stress Scale (0.464), and the Academic Burnout Scale (0.364). Furthermore, the square roots of the AVE for the Social Networking Site Addiction Scale, Smartphone Addiction Scale, Academic Stress Scale, and Academic Burnout Scale are all greater than their respective correlation coefficients with the SVAS-MSS. This demonstrates that the SVAS-MSS is capable of distinguishing itself from these related yet distinct constructs, thereby exhibiting robust discriminant validity.

**Table 7:** The AVE and √AVE between the SVAS-MSS and the criterion scales (*n* = 1009).

	AVE	√AVE	1	2	3	4	5
1 SVAS-MSS	0.556	0.746	–				
2 Social Networking Site Addiction	0.382	0.618	0.532**	–			
3 Smartphone Addiction	0.493	0.702	0.690**	0.645**	–		
4 Academic Stress	0.491	0.701	0.464**	0.375**	0.517**	–	
5 Academic Burnout	0.388	0.623	0.364**	0.356**	0.431**	0.433**	–

*p<0.05, **p<0.01, ***p<0.001.

#### Criterion validity

To evaluate the criterion validity of the SVAS-MSS, the Social Networking Site Addiction Scale, Smartphone Addiction Scale, Academic Stress Scale, and Academic Burnout Scale were selected as benchmark instruments. The selection of these scales is underpinned by their theoretical and behavioral relevance to short video addiction, particularly in the context of the distinct developmental characteristics of middle school students.

Primarily, middle school students are navigating a pivotal phase of self-identity exploration and rapidly evolving social needs. During this developmental stage, students increasingly depend on social networks and smartphones to establish and sustain interpersonal relationships, thereby satisfying their escalating demands for social interaction. This heightened engagement with social networks and smartphones creates a conducive environment for the utilization of short video platforms, as many short video applications integrate social functionalities. Consequently, students engage in social interactions concurrently with short video consumption, enhancing both the appeal and frequency of short video use. This multifunctional usage pattern elevates the risk of addictive behaviors. Therefore, the Social Networking Site Addiction Scale and Smartphone Addiction Scale effectively capture the behavioral patterns and psychological traits associated with short video addiction.

Secondly, the substantial academic pressures characteristic of the middle school phase may lead students to seek emotional relief and escapism through excessive short video consumption, thereby fostering addictive behaviors. In this regard, the Academic Stress Scale and Academic Burnout Scale are instrumental in assessing the relationship between short video addiction and academic-related psychological states.

It is hypothesized that the SVAS-MSS will demonstrate moderate to high positive correlations with both the Social Networking Site Addiction Scale and Smartphone Addiction Scale, given the significant overlap in device usage frequency and time investment inherent in these addictive behaviors. Additionally, significant positive correlations are anticipated between the SVAS-MSS and both the Academic Stress Scale and Academic Burnout Scale, reflecting the tendency of middle school students to utilize short videos as a coping mechanism for academic stress.

Empirical findings, as delineated in Table 7, reveal that the correlation coefficients between the SVAS-MSS and the benchmark scales range from 0.356 to 0.690. These significant correlations not only substantiate the theoretical expectations but also affirm the applicability and efficacy of the SVAS-MSS within the middle school demographic, thereby indicating robust criterion validity.

### Measurement invariance by gender

To examine whether the SVAS-MSS can be compared across genders, a measurement invariance analysis was conducted.

#### Single-group confirmatory factor analysis (baseline model)

A single-group confirmatory factor analysis was conducted on Sample 4. The results, presented in Table 8, show that the five-factor model demonstrated the following fit indices across different groups (overall, male, and female middle school students): CFI and TLI ranged from 0.914 to 0.970, RMSEA ranged from 0.047 to 0.074, and SRMR ranged from 0.031 to 0.044. All indices met the criteria for good model fit, indicating that the five-factor model of short video addiction is appropriate for both male and female groups. These results suggest that the scale is suitable for gender invariance testing.

**Table 8:** Fit indices for the baseline model of the scale (*n* = 1009).

Parameters	*n*	χ^2^/df	CFI	TLI	RMSEA (90% CI)	SRMR
Total sample	1009	3.275	0.970	0.960	0.047 [0.041, 0.054]	0.031
Male sample	554	2.360	0.965	0.954	0.050 [0.040, 0.059]	0.033
Female sample	455	3.519	0.935	0.914	0.074 [0.065, 0.084]	0.044

#### Gender invariance testing

A gender invariance test was conducted on Sample 4, with results shown in Table 9. The CFI and TLI indices for the three models ranged from 0.934 to 0.951, the RMSEA indices ranged from 0.058 to 0.062, and the SRMR indices ranged from 0.038 to 0.042, all meeting the criteria for good model fit. Additionally, pairwise comparisons between the models showed that ∆CFI was less than 0.01, ∆TLI was less than 0.01, and ∆RMSEA was less than 0.015. These findings indicate that the SVAS-MSS for Junior High School Students demonstrates gender invariance, making it suitable for cross-group comparisons between male and female students.

**Table 9:** Model fit indices based on gender variable (*n* = 1009).

Model	CFI	TLI	RMSEA (90% CI)	SRMR
Configural invariance	0.950	0.934	0.062 [0.056, 0.069]	0.038
Weak invariance	0.950	0.938	0.060 [0.054, 0.067]	0.042
Strong invariance	0.951	0.942	0.058 [0.052, 0.064]	0.042

## Discussion

In today’s digital era, short video platforms have become an integral part of middle school students’ daily lives, offering engaging, easily consumable content that aligns with their developmental need for stimulation. However, the addictive nature of these platforms has raised concerns about their impact on students’ psychological and behavioral well-being. Excessive use of short videos can lead to negative outcomes, including compulsive viewing, behavioral dysregulation, Attention Concentration Difficulties, interpersonal challenges, and difficulties in academic adaptation. Despite growing attention to the effects of digital media, the specific impact of short video addiction on middle school students remains underexplored. This study addresses this gap by developing and validating a reliable scale to assess short video addiction in this age group, focusing on both psychological and behavioral manifestations.

### Core manifestations of short video addiction among middle school students

The qualitative findings from in-depth interviews (Study 1) revealed key psychological motivations and behavioral patterns associated with short video use among middle school students. The primary reasons for using short videos included escaping academic pressures and engaging in social interactions, with increased usage on weekends. These motivations and behaviors align with existing research on the addictive nature of short video platforms^5,8,10,14,19^. Based on these findings, five core manifestations of short video addiction were identified: interpersonal relationship issues, learning adaptation challenges, Attention Concentration Difficulties, behavioral dysregulation, and compulsive viewing behaviors. These manifestations reflect the significant psychosocial and academic impact that short video addiction can have on middle school students, consistent with research on the negative outcomes of excessive online engagement^5,10,14,33–38^.

### Psychometric properties of the SVAS-MSS

Study 2 building upon the interview findings from Study 1. The SVAS-MSS was developed through rigorous item analysis, exploratory factor analysis (EFA), and confirmatory factor analysis (CFA). Results from both EFA and CFA demonstrated that the scale has a stable five-factor structure, encompassing procrastination behavior, strained social relationships, deteriorating social status, Attention Concentration Difficulties, and Impaired Control over Short Video Use, 15 items were retained. These findings are consistent with previous studies that have identified similar dimensions of internet and short video addiction^5,10^. The scale demonstrated strong internal consistency (Cronbach’s α = 0.905) and test-retest reliability (ICC = 0.788), indicating that it is a reliable tool for assessing short video addiction.

The significant positive correlations between the SVAS-MSS and both the Academic Stress Scale and Academic Burnout Scale highlight a concerning relationship between short video addiction and academic challenges. As short video consumption increases, it seems to exacerbate academic stress and contribute to feelings of burnout, which is particularly troubling for middle school students facing both cognitive and emotional changes as well as rising academic demands^14,20^.

In addition, the significant negative correlations observed between the SVAS-MSS and the Social Networking Site Addiction Scale and Smartphone Addiction Scale suggest that short video addiction may be part of a broader pattern of digital media overuse. Excessive engagement with short videos, like other forms of digital addiction, can disrupt time management and reduce students’ ability to focus on school-related tasks. This results in increased academic pressure and frustration, leading to burnout^39,40^.

These findings point to a larger issue of digital media addiction, where multiple forms of screen time—whether through short videos, social media, or smartphones—are all contributing factors to academic and psychological stress. Addressing short video addiction, therefore, requires a comprehensive approach that considers the broader context of digital media use and its impact on students’ mental well-being and academic performance.

### Gender measurement invariance

Measurement invariance was tested across gender, with results indicating that the scale is applicable to both male and female middle school students. This suggests that the identified dimensions of short video addiction are consistent across demographic categories, which enhances the generalizability of the findings. These results align with prior studies on internet addiction, which have similarly reported no significant gender differences in addiction patterns^41–43^.

### Implications

The SVAS-MSS provides a valuable tool for assessing and addressing the specific characteristics of short video addiction in middle school students. It is distinct from existing general internet addiction measures in that it focuses on the unique behavioral and psychological aspects of short video consumption. This targeted approach is essential for developing effective intervention strategies for students at risk of addiction. Schools and families can use the scale to identify early warning signs and implement preventative measures to mitigate the negative impact of short video addiction.

### Limitations and future directions

This study has several limitations. First, the sample was drawn from a specific demographic group (middle school students), which may limit the generalizability of the findings to other age groups or populations. Second, the reliance on self-report data introduces potential biases, such as social desirability or recall inaccuracies. Third, while the SVAS-MSS demonstrated strong psychometric properties, further validation across diverse cultural and socioeconomic contexts is necessary to ensure its broader applicability. A critical oversight is the absence of a validated cut-off score, which limits the scale’s clinical utility in distinguishing normative use from pathological addiction. Without empirically derived thresholds, educators and clinicians lack clear benchmarks to identify at-risk individuals or prioritize interventions.

Future research should address these limitations while expanding the scope of inquiry. Longitudinal studies are needed to examine the long-term effects of short video addiction on academic performance, mental health, and social functioning, as well as the efficacy of targeted interventions. Additionally, while this study focused on negative outcomes, exploring potential positive aspects of short video use—such as its role in fostering social connections, creative expression, or educational engagement—could provide a more balanced understanding of this medium. Establishing cut-off scores for the SVAS-MSS must be prioritized through methods such as receiver operating characteristic (ROC) curve analysis, comparing scale scores against clinical diagnoses or functional impairment criteria. This would enhance the tool’s practical relevance for screening and intervention. Finally, cross-cultural validation studies and mixed-method approaches (e.g., integrating behavioral data with self-reports) could strengthen the scale’s robustness and generalizability. Addressing these gaps will bridge theoretical measurement with real-world application, positioning the SVAS-MSS as a cornerstone for addressing short video addiction in adolescents.

## Conclusion

This study developed and validated the Short Video Addiction Scale for Middle School Students (SVAS-MSS) to address the rising public health concern of problematic short video use among adolescents. Through qualitative interviews and psychometric analyses with 1,492 participants, the scale identified five core dimensions: Academic Procrastination, Interpersonal Strain, Social Communication Difficulties, Attention Concentration Difficulties, and Impaired Control. Demonstrating high reliability (α = 0.900) and robust validity, the SVAS-MSS fills a critical gap by providing the first age-specific tool to assess short video addiction. Its structured framework enables educators and clinicians to identify at-risk students early, guiding targeted interventions to mitigate adverse academic, social, and psychological impacts. This advancement supports healthier digital habits and underscores the need for continued research into adolescent-specific behavioral measurement tools.

## Electronic supplementary material

Below is the link to the electronic supplementary material.

Supplementary Material 1

## Data Availability

The datasets used and/or analyzed during the current study available from the corresponding author on reasonable request.

## Declarations

### Pre-registered

The study was not pre-registered.
