# Supplementary material for: Short video addiction scale for middle school students: development and initial validation
Source: Sci Rep. 2025 Mar 22;15:9903. doi: 10.1038/s41598-025-92138-x (PMC11929814; doi:10.1038/s41598-025-92138-x)
Supplement: Supplementary file 1 — Supplementary Material 1 [file 41598_2025_92138_MOESM1_ESM.docx]

**Supplementary Material**

**Supplementary Material 1: Interview Guide on Manifestations of Short Video Addiction among Middle School Students Interview Guide Overview**

The interview guide encompasses the following questions, with flexibility for follow-up inquiries based on the respondent's answers:

***Section 1: Recording Consent and Topic Introduction***

1. Hello, we will focus on the topic of short video usage today. You are only required to respond to my questions.
2. Please feel free to share your genuine thoughts and feelings. Since our conversation will later be analyzed, may I have your permission to record it?
3. To begin, could you tell me when you typically use your phone, and what activities you usually engage in while using it?
4. Approximately how much of this time is spent on short videos?
5. Which platforms do you use to watch short videos? When did you first start engaging with short videos?
6. How much time do you spend on short videos during weekdays versus weekends?
7. Do you create short videos yourself?

***Section 2: Manifestations of Short Video Addiction***

1. Psychological and Behavioral Symptoms of Compulsive Short Video Viewing:

a) Have you ever experienced a situation where you did not want to watch short videos but found yourself unable to stop?

b) Have you ever continued watching short videos despite feeling sleepy and wanting to go to bed?

c) Have you ever kept watching short videos even when your eyes were sore?

d) What is your emotional experience when you want to stop watching short videos but find yourself unable to control it?

1. Behavioral Disruptions Resulting from Short Video Usage:

a) Has watching short videos led to any adverse consequences for you?

b) Have you been late, skipped classes, or missed important events because of watching short videos?

1. Attention Difficulties Due to Short Video Usage:

a) Do you find yourself distracted by thoughts of short video content while in class or doing homework?

b) Have you forgotten to do things you planned because of watching short videos?

c) Do you feel difficulty engaging in tasks, such as completing homework, after watching short videos?

1. Interpersonal Issues Arising from Short Video Usage:

a) Has watching short videos affected your relationships with your parents or classmates?

b) Have you had arguments with your parents due to watching short videos?

c) Have you experienced unpleasant situations with classmates because of your short video usage?

1. Academic Adjustment Difficulties Related to Short Video Usage:

a) Has watching short videos impacted your academic performance?

b) Do you have the urge to watch short videos while studying?

c) Have you experienced situations where you were unable to complete assignments due to watching short videos?

***Section 3: The Relationship Between Short Video Addiction and Academic Pressure***

1. When you feel pressured by academic demands, what methods do you use to alleviate this stress? (If short videos are mentioned, proceed to question 3.)
2. Do you use watching short videos as a means to relax?
3. Why do you think watching short videos helps you relieve academic stress?

***Section 4: Additional Open-Ended Questions***

1. In your opinion, what level of short video usage constitutes addiction?
2. How do those around you perceive your short video usage?
3. Do you believe you have a tendency towards short video addiction? Why or why not?
4. Are there individuals in your environment who exhibit signs of short video addiction? What are their behaviors?
5. Do you have any thoughts or questions regarding our interview?

**Supplementary Material 2: Results of Interview - Three-Level Coding**

| **Three-Level Coding** | **Two-Level Coding** | **One-Level Coding** | **Example (Partial)** |
| --- | --- | --- | --- |
| Issues related to interpersonal relationships caused by the use of short videos (24) | Include relationship difficulties with parents (11) | Perceiving a generational communication gap with parents (3) | While browsing videos online, I come across some popular terms that my parents might not know, leading to communication barriers with them. |
|  |  | Arguments with parents due to watching short videos (8) | My parents may feel that I spend too much time on my phone and might view this behavior negatively, resulting in frequent arguments. |
|  | Relationship issues with friends or classmates (13) | Inappropriate use of memes (5) | For example, during a break, if a classmate says something similar to a meme from the internet, other classmates might use that meme to give the classmate a nickname. |
|  |  | Delaying or forgetting commitments (1) | When a friend invited me to play basketball, I initially intended to go, but I became so engrossed in watching videos that I missed the commitment to my friend. |
|  |  | Adopting offensive and insulting language from short videos (7) | Some boys might adopt inappropriate language from short videos and make lewd jokes towards girls. |
| Learning adaptation issues caused by the use of short videos (43) | Delaying or failing to complete assignments (16) | Forgetting to do assignments (5) | For instance, when I need to complete assignments or attend online classes on weekends and must use my phone to finish the work, I often start by watching short videos. After spending a considerable amount of time on them, I then remember that I need to do my assignments. |
|  |  | Watching short videos during study sessions (5) | If you need to do homework but find your mind involuntarily drifting to funny videos you watched earlier, it can prevent you from completing your assignments and studying effectively, leading you to compulsively return to your phone to watch more videos. |
|  |  | Inability to finish assignments (6) | What was intended as a brief video session can turn into an afternoon of browsing, thereby consuming the time allocated for completing assignments. Such situations do occur. |
|  | Decline in academic performance (5) | Personal perception of a decline in grades due to watching short videos (2) | When I was in elementary school, my grades were quite good. However, when I discovered short videos in fifth grade, I started using my grandmother’s phone daily to watch them. Consequently, I became unwilling to do my homework, and my grades declined. |
|  |  | Parents' belief that watching short videos affects academic performance (3) | When my parents see me watching short videos, they associate it with the decline in my academic performance. |
|  | Impact on classroom engagement (12) | Staying up too late the night before affects the following day’s classroom performance (8) | The next day, my mental state is poor, and I become drowsy during class, with diminished concentration, sometimes even falling asleep during lessons. |
|  |  | Thinking about short video content during class (4) | During class, I find myself thinking about popular internet memes. |
|  | Impairment of learning skills (10) | Deterioration of verbal expression skills (2) | This deterioration affects overall learning abilities and may also impair verbal expression skills. |
|  |  | Decline in memory retention (4) | Extensive binge-watching of short videos can lead to a noticeable decline in memory retention the following day. |
|  |  | Decline in cognitive abilities (4) | Excessive video consumption, combined with reduced cognitive engagement during viewing, can result in diminished mental sharpness. |
| Attention disorders caused by the use of short videos (27) | Narrow attention span (5) | Becoming so engrossed in watching that one becomes oblivious to others speaking (3) | Being so absorbed in viewing that it feels as if one is unable to hear anything else. |
|  |  | Inability to put down short videos to engage in other activities (2) | At home, when I need to complete certain tasks, if my parents ask me to do something, I might procrastinate, saying I'll do it later. |
|  | Distracted attention (14) | Distraction during homework (6) | While doing homework, certain stimulating words can trigger thoughts of memes. |
|  |  | Distracted attention during class (8) | During class, I find myself thinking about popular internet memes. |
|  | Reduced attention (8) | Difficulty in maintaining focus during study (5) | I primarily find it challenging to calm my mind for studying. |
|  |  | Lack of patience for tasks (3) | Short videos highlight only the most exciting parts of movies, leading to a lack of patience; I tend to fast-forward through TV shows and flip through books rapidly. |
| Behavioral dysregulation caused by the use of short videos (48) | Loss of control over speech and behavior (32) | Inability to refrain from using memes (10) | Constantly using popular internet memes in conversation. |
|  |  | Interrupting the teacher during class when encountering content related to short videos (11) | During a math class, when the teacher was explaining something, a student interrupted by shouting out an internet meme. |
|  |  | Inability to resist mimicking actions from short videos (9) | Sometimes, we imitate popular online actions, which can influence our everyday behavior. |
|  |  | Inability to stop watching short videos (2) | I intended to finish watching this and then leave, but ended up watching for an extended period. |
|  | Emotional dysregulation (3) | Irritability (2) | He tends to use memes in his speech, which contributes to his irritability. |
|  |  | Increased anxiety due to an abundance of fragmented information (1) | An accumulation of fragmented information in the mind may lead to increased anxiety. |
|  | Loss of control over usage duration (13) | Excessive usage during the day (6) | What was intended as a brief session can extend to three or four hours, or an entire afternoon. |
|  |  | Watching short videos at night, leading to late sleep (7) | The content of short videos is so engaging that I find it hard to stop watching, leading to very late nights. |
| Compulsive watching of short videos (13) | Compulsive behavior (12) | Desiring sleep but unable to stop watching (4) | My mind tells me that it’s too late to continue watching short videos, yet my hands keep scrolling uncontrollably. |
|  |  | Inability to refrain from watching while eating (3) | Even though I realize halfway through that I should be eating, I am unable to stop watching. |
|  |  | Inability to stop watching even when feeling annoyed (1) | I keep scrolling through short videos, and even if I start feeling annoyed, I may watch just 10 seconds of a 30-second video before immediately moving on to the next. |
|  |  | Inability to control watching while doing homework (4) | Even though I realize it's getting late and I need to start on my homework, I struggle to exit the short videos and cannot control myself. |
|  | Compulsive thinking (1) | Mental exhaustion due to the belief that watching short videos is inappropriate (1) | I keep thinking about why I watched so many videos today when I could have finished my homework and gone to bed earlier, resulting in mental exhaustion. |

**Supplementary Material 3: Preliminary Scale on Short Video Addiction Among Middle School Students**

| **Factor** | **Item** |
| --- | --- |
| Interpersonal relationship difficulties triggered by short video usage (9) | 1.I have hurt friends/classmates by using popular memes (imitating their language or actions) from short videos. |
|  | 6.I have delayed or missed activities with friends/classmates because of watching short videos. |
|  | 11.Family/friends/classmates say that I have ignored what they said because of watching short videos. |
|  | 16.I argue with my parents/friends because of watching short videos. |
|  | 20.Parents/friends express dissatisfaction or complaints about my use of short videos. |
|  | 24.Virtual social circles in short videos have made my real-life relationships more distant. |
|  | 28.I ignore my friends around me in order to get likes and comments on short videos. |
|  | 32.During conversations with family/friends/classmates, I have ignored what they say because of watching short videos. |
|  | 36.Watching short videos has reduced my communication with family/friends. |
| Learning adaptation challenges resulting from short video usage (12) | 2.I get distracted during class because I am thinking about the content of short videos. |
|  | 7.Staying up late watching short videos has made it difficult for me to stay awake and focused in class. |
|  | 12.Parents/friends believe that my academic performance has declined due to watching short videos. |
|  | 17.Watching short videos has led to a decline in my academic performance. |
|  | 21.I feel that my cognitive abilities have declined due to watching short videos. |
|  | 25.I feel that my memory has declined due to watching short videos. |
|  | 29.I feel that my verbal expression skills have declined due to watching short videos. |
|  | 33.I watch short videos while doing homework, which extends the time I spend on homework. |
|  | 37.I forget my original study plan because of watching short videos. |
|  | 3.I delay starting my homework because of watching short videos. |
|  | 8.I have lost interest in studying because of watching short videos. |
|  | 13.Short videos have made me feel bored with studying. |
| Attention disorders caused by short video usage;  (6) | 18.After watching short videos, I find it difficult to focus on other tasks. |
|  | 22.After watching short videos, I find it hard to regain focus for studying. |
|  | 26.Watching short videos makes it difficult for me to focus on tasks that require prolonged thinking or studying. |
|  | 30.Class content reminds me of the short videos I have watched. |
|  | 34.While watching short videos, I forget about the surrounding time and environment. |
|  | 38.I cannot immediately stop watching short videos to do other tasks. |
| Uncontrolled behaviors induced by short video usage (8) | 4.I spend more time watching short videos than originally planned. |
|  | 9.I cannot control the time I spend watching short videos, leading to staying up too late. |
|  | 14.I cannot resist using popular memes (imitating language or actions) from short videos. |
|  | 19.When I see content similar to short videos in class, I cannot help but discuss it with classmates. |
|  | 23.When parents or teachers stop me from watching short videos, I feel angry or resistant. |
|  | 27.The fragmented information from short videos makes me feel anxious. |
|  | 31.Watching short videos has made me increasingly impatient with other activities (e.g., conversation, reading). |
|  | 35.I become frustrated when I cannot find content I am interested in while watching short videos. |
| Psychological and behavioral manifestations of compulsive short video viewing (4) | 39.I have tried to control/stop my short video watching behavior but have been unsuccessful. |
|  | 5.I cannot stop watching short videos even when I have other important tasks to do. |
|  | 10.Even when I know I should be doing homework/eating/sleeping, I cannot stop watching short videos. |
|  | 15.I feel guilty about watching short videos but still want to continue watching them. |

**Supplementary Material 4: Official Scale on Short Video Addiction Among Middle School Students**

| **Factor** | **Item** |
| --- | --- |
| Social Communication Difficulties | 1. Watching short videos has made me increasingly impatient with other activities (such as conversations, reading, etc.). |
|  | 2. While talking with family/friends/classmates, I ignore what they are saying because I am busy watching short videos. |
|  | 3. I have reduced communication with my family/friends/classmates because of watching short videos. |
|  | 4. I feel that my language expression ability has declined due to watching short videos. |
| Academic Procrastination | 5. I delay starting my homework because of watching short videos. |
|  | 6. I watch short videos while doing homework, which prolongs the time needed to complete it. |
|  | 7. I spend more time watching short videos than I initially planned. |
| Attention Concentration Difficulties | 8. After watching short videos, I find it hard to focus on other tasks. |
|  | 9. After watching short videos, I find it difficult to refocus on studying. |
|  | 10. Watching short videos makes it hard for me to concentrate on tasks that require prolonged thinking or studying. |
| Interpersonal Strain | 11. My parents/friends/classmates think that my academic performance has declined because of watching short videos. |
|  | 12. My parents/friends have expressed dissatisfaction or complaints because I watch short videos. |
|  | 13. I have argued with my parents/friends because of watching short videos. |
| Impaired Control over Short Video Use | 14. I have tried to control/stop my short video watching habits, but I have failed. |
|  | 15. I cannot immediately stop watching short videos to do other things. |
